# Development of a teledermatopathology consultation system using virtual slides

**DOI:** 10.1186/1746-1596-7-177

**Published:** 2012-12-13

**Authors:** Ikunori Nakayama, Tsubasa Matsumura, Akihisa Kamataki, Miwa Uzuki, Kenji Saito, James Hobbs, Toshihide Akasaka, Takashi Sawai

**Affiliations:** 1 Department of Dermatology, Course of Integrated Medicine, Graduate School of Medicine, Osaka University, Suita, Osaka, Japan; 2 Division of Leading Pathophysiology, Department of Pathology, Iwate Medical University School of Medicine, Yahaba, Iwate, Japan; 3 Department of Dermatology, Iwate Medical University School of Medicine, Morioka, Iwate, Japan; 4 Information Center for Iwate Medical University, Morioka, Iwate, Japan; 5 Department of English, Center for Liberal Arts and Sciences, Iwate Medical University, Yahaba, Iwate, Japan

**Keywords:** Telepathology, Teledermatology, Consultation, Virtual slide, Whole slide image

## Abstract

**Background:**

An online consultation system using virtual slides (whole slide images; WSI) has been developed for pathological diagnosis, and could help compensate for the shortage of pathologists, especially in the field of dermatopathology and in other fields dealing with difficult cases. This study focused on the performance and future potential of the system.

**Method:**

In our system, histological specimens on slide glasses are digitalized by a virtual slide instrument, converted into web data, and up-loaded to an open server. Using our own purpose-built online system, we then input patient details such as age, gender, affected region, clinical data, past history and other related items. We next select up to ten consultants. Finally we send an e-mail to all consultants simultaneously through a single command. The consultant receives an e-mail containing an ID and password which is used to access the open server and inspect the images and other data associated with the case. The consultant makes a diagnosis, which is sent to us along with comments.

Because this was a pilot study, we also conducted several questionnaires with consultants concerning the quality of images, operability, usability, and other issues.

**Results:**

We solicited consultations for 36 cases, including cases of tumor, and involving one to eight consultants in the field of dermatopathology. No problems were noted concerning the images or the functioning of the system on the sender or receiver sides. The quickest diagnosis was received only 18 minutes after sending our data. This is much faster than in conventional consultation using glass slides. There were no major problems relating to the diagnosis, although there were some minor differences of opinion between consultants. The results of questionnaires answered by many consultants confirmed the usability of this system for pathological consultation. (16 out of 23 consultants.)

**Conclusion:**

We have developed a novel teledermatopathological consultation system using virtual slides, and investigated the usefulness of the system. The results demonstrate that our system can be a useful tool for international medical work, and we anticipate its wider application in the future.

**Virtual slides:**

The virtual slides for this article can be found here: http://www.diagnosticpathology.diagnomx.eu/vs/1902376044831574

## Background

Skin diseases vary widely from conditions that can interfere with social activity because of cosmetic disorders, such as acne and alopecia, to diseases that affect patient prognosis and quality of life, such as malignant melanoma or basal cell cancer, as well as various chronic inflammatory diseases. Achieving an accurate diagnosis and deciding on an appropriate procedure for therapy often requires microscopic as well as macroscopic findings, but very few dermatologists in Japan engage in histopathological diagnosis, and very few pathologists specialize in dermatopathology [1,2]. Furthermore, given the disproportionate availability of physicians and medical facilities between densely populated urban areas and more sparsely populated areas such as mountain or coastal areas, access to dermatological care is often woefully inadequate for achieving accurate diagnoses. To compensate for this in Japan, conventional consultation based on the delivery of glass slides has been available. This is a labor-, time-, and cost-intensive system, however, because many slides must be prepared and then delivered to experts. Delays in diagnosis and slide loss or damage are also major concerns.

Recently, in an alternative consultation system, image data of digitalized pathological findings have been transmitted to remote experts via the Internet [3,4], and also via mobile phone with static images and short movies [5].

However, compared to actual microscopic observation, the visual field, magnification and focus of the static images cannot be adjusted, making this more time-consuming and stressful than routine non-telepathological diagnosis [6,7].

The use of virtual slide (VS), especially recent whole slide image (WSI) makes it possible to select the visual field and magnification, and even adjust focus, resulting in diagnostic accuracy comparable to that achieved by conventional optical microscopy [7-14]. The use of VS has contributed to a gradual increase in consultations in dermatopathology as well as in other fields. However, the consultation system has continued to rely on the traditional communication tools of e-mail and facsimile. This system does not lend itself to the systematic organization and recording of consultation case details and diagnoses, and results in delays finding pathologists in specific fields suited to particular cases. Furthermore, it is not easy to compare the diagnosis received from one consultant with those returned by others and with previous cases.

In this study we developed a consultation system combining VS with a web application offering access to many consultants in a range of professional fields. The effectiveness of the consultation system for dermatological cases was compared with that of conventional consultations using glass slides, and with conventional telepathology using static images.

This study focused not on the accuracy of diagnoses obtained using VS, which has been fully evaluated in recent studies [7-14], but on the performance and future potential of this new consultation.

## Methods

### The consultation system

**Figure 1:**
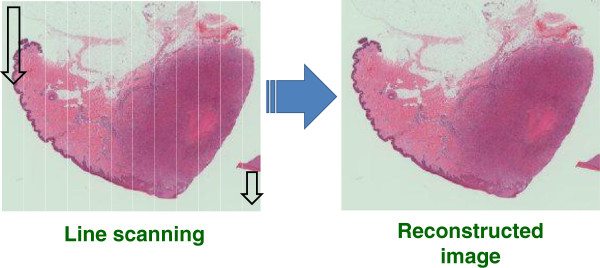
**Virtual slide image.** JPEG images produced by line scanning were reconstructed in the form of digital data. Images were always just in focus, even when changing magnification.

The system is composed of telecommunication lines, a VS system, and a public web server for consultations. Telecommunication lines included the local area network at Iwate Medical Uniceristy (100BASE-TX), used for actions such as uploading VS images, as well as B Flet’s® (best-effort 100 Mbps) fiber optic lines; Nippon Telegraph and Telephone Corporation EAST (NTT EAST; Tokyo, Japan) lines were used for external communication for transmission and reception via the public server. A Scan Scope CS® scanner (Aperio Technologies, Vista, CA) was used for the VS system. Resolution could be set to an objective lens magnification of 20× (0.50 μm/pixel) or 40× (0.25 μm/pixel). Diagnosis could usually be achieved at 20× magnification, but 40× magnification was used for scanning when detailed observation of cellular morphology was required for distinction of poorly differentiated or undifferentiated malignancies, or leukemia. The computer was a Workstation 4300 (Hewlett-Packard Development Company L.P., Palo Alto, CA), equipped with the Windows XP® operating system, an Intel Pentium D® 3.20 GHz CPU, and an mvBlue Fox (Matrix Vision GmbH, Oppenweiler, Germany) graphics board with 1.64 GB RAM and an 800 GB HDD. VS images were displayed by reconstructing scanned JPEG 2000 digital images (Figure 1). Images that were always in focus were thus displayed on a monitor, and the visual field and magnification could be adjusted in much the same way as with a microscope.

**Figure 2:**
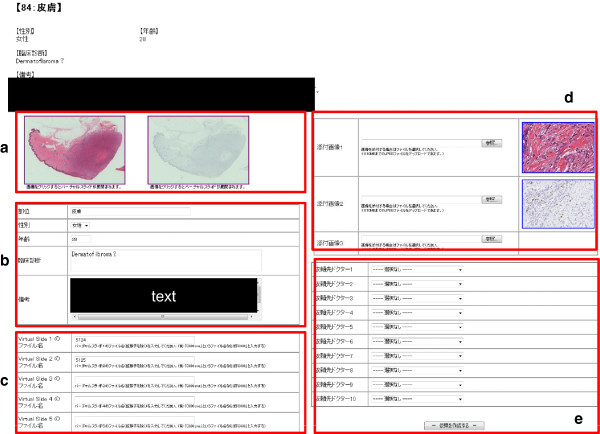
**Ordering page. a**) Click the thumbnail image of a VS to display the image. **b**) Input patient information (sex, age, region, clinical diagnosis, comments). **c**) Select VS to send (up to 5 slides). **d**) Add static images such as previous histological figures, X-ray films or macroscopic photographs necessary for patient diagnosis (up to 3). **e**) Select consultants from the list (up to 10).

A ProLiant Server DL120 G6 (Hewlett-Packard) with a FreeBSD operating system and an Intel Xeon® X3430 2.4 GHz CPU, with 4 GB RAM and a 640 GB HDD, was used as the public web server for consultations. This included a database function for managing digital images and patient data (such as case number and medical history), and consultation-related web application functions, such as order input, digital image display, e-mail notifications from the sender site, response input from the receiver site (consultant), and response verification from the sender. This allowed the series of actions required for carrying out consultations to be implemented through a web browser not dependent on a particular operating system or dedicated software. As an additional function, VS images could be displayed up to five at a time, and static images (macroscopic photographs, X-rays, medical charts, and other documents needed for diagnosis) could be displayed up to three at a time. In addition, up to 10 pre-registered diagnosticians could be selected, and a consultation request could be sent to all in a single action (Figure 2).

To protect patient identity and personal details, information was limited to age, region, and clinical diagnosis.

### Questionnaire survey about our system

Forty-two pathologists and dermatologists were surveyed by questionnaire on items such as the content, operability, and image quality of the system. Individual e-mail requests to fill out the questionnaire were sent, and responses were returned by either e-mail or fax. The following items were surveyed:

1) The percentage of consultation cases among routinely diagnostic cases

2) Operability of VS displayed on the web browser;

3) Image quality, especially for diagnosis; and

4) Convenience and usability of this system for supporting their work.

## Results

### The consultation system

Our system was used to consult 10 pathologists or dermatologists about skin diseases. Diagnostic consultations were requested in 36 cases (13 men, 23 women) (Table 1). Generally the data volume of VS was proportional to the scanning area and resolution, usually at about 150–300 MB with an area of about 15×15 mm^2^ and a resolution corresponding to an objective lens magnification of 20 power. Consultations covered neoplastic diseases that were identified or determined to be benign or malignant in 20 cases, and non-neoplastic diseases not amenable to definitive diagnosis involving rare inflammatory, degenerative, or congenital disease in 16 cases. In 24 cases 1 VS image was referred for diagnosis, in 2 cases 2 slides, and in 10 cases 3 or more slides. Macroscopic photographs were attached in 2 cases. Other laboratory data were also sent in 1 case. Most consultations were with dermatologists in Iwate Medical University Hospital and in other universities or medical facilities throughout Japan. Thirty cases were sent to 1 consultant, 1 case to 3, 1 case to 4, 2 cases to 5, and 2 cases to 8 consultants. The fastest response time was 18 minutes. Some cases were not answered by all pathologists. All pathological diagnoses from consultants were relayed with their comments to clinicians. However, after considering all diagnoses and comments, the final responsibility for critical pathological diagnosis relating to patients’ therapy usually rested with the sender pathologist. Cases in which diagnoses differed between multiple consultants were reviewed and left to the discretion of the requesting side.

**Table 1:** List of consultations From July 1, 2011 to March 19, 2012, this system was used for 36 consultations (13 men, 23 women) in Japan

**No**	**Sex**	**Age**	**Region**	**The number of VS images (static image)**	**Clinical diagnosis**	**Pathological diagnosis**	**The number of consultants (no response)**	**Response time (D/Hr/Min)**
1	M	85	Face	3	Skin tumor, recurrent	Epithelioid sarcoma	1	11/20/14
2	F	11	Nose	1	Rash on nose	Fibrofolliculoma (Trichodiscoma)	1	2/20/43
3	M	81	Skin	3	Mastocytoma	Nevus pigmentosus	1	5/3/26
4	F	73	Buttocks	4	Skin tumor	Congenital intradermal nevus	1	3/0/50
5	F	71	Lower leg	1	Subcutaneous tumor	Eccrine poroma	1	1/17/22
6	F	42	Left inguinal lymph node	1	Subcutaneous tumor	Atypical lymphoproliferative disorder	1	0/16/36
7	M	68	Back	1	Disseminated granuloma annulare	Disseminated granuloma annulare	1	0/19/17
8	F	14	Right upper arm	1	Nevus	Pigmented nevus	1	4/19/53
9	M	85	Face	4	Subcutaneous tumor	Epithelioid sarcoma	1	3/2/25
10	F	29	Face (cheeks)	1	Nevus	Compound nevus	5	0/21/6
11	F	75	Conjunctiva	1	Subcutaneous tumor	Sebaceoma	1	0/6/25
12	F	46	Thigh	5	Subcutaneous tumor	Dermatofibrosarcoma protuberans	1	0/1/22
13	M	64	Head (crown)	1	Subcutaneous tumor	Metastatic skin cancer	1	0/17/08
14	F	96	Left knee	3 (1)	Squamous cell carcinoma	Seborrheic keratosis	1	0/1/22
15	M	36	Post-auricular	1	Fibrous histiocytoma	Kimura’s disease	1	0/1/33
16	M	74	Lower leg	1	Erythema nodosum	Dermopanniculitis	1	0/5/9
17	M	48	Right heel	1	Bowen disease	Verruca plantaris with contact dermatitis	1	0/2/11
18	F	82	Right outer thigh	1	Bowen disease	Clear cell basal cell carcinoma	1	0/22/39
19	F	67	Right inguinal region	1	Melanocytic nevus	Letigo maligna melanoma	1	0/22/27
20	M	29	Right cheek	3	Epidermal cyst	Granuloma, post panniculitis	1	0/22/19
21	F	68	Entire body	2	Vesicular disease	Erythema multiforme	1	0/3/13
22	M	32	Forehead	1	Epidermal cyst	Proliferating trichilemmal cyst	1	0/0/18
23	F	82	Face	1	Facial tumor	Irritated seborrheic keratosis	1	4/3/35
24	M	84	Face (right cheek)	2 (1)	Aggregated milia	Favre-Racouchot syndrome	1	1/1/50
25	F	26	Left middle of toe	1	Melanocytic nevus	Malignant melanoma *in situ*	1	0/2/17
26	F	57	Face	1	Subcutaneous tumor	Trichoblastoma partially with sebaceous differentiation	1	0/0/31
27	F	38	Both feet	1	Atypical nevus	compound nevus	1	0/4/18
28	M	71	Neck	1	Skin tumor on neck	eccrine poroma	1	0/6/1
29	F	61	Back	3	Skin tumor on back	Dermatofibrosarcoma	8 (1)	0/1/5
30	F	83	Left forearm	3	Skin malignancy	Malignant cellular blue nevus	1	0/0/51
31	M	34	Right sole	1 (3)	Tumor on right sole	Epithelioid sarcoma	8 (1)	0/0/36
32	F	59	Skin of lower jaw	1	Subcutaneous tumor	Extraocular sebaceous carcinoma	4	0/18/38
33	F	28	Right forearm	3	Dermatofibroma	Dermatofibroma	3	0/0/22
34	F	68	Right eyelid	1	Cutaneous horn	Lichen planus-like keratosis	1	0/18/44
35	F	74	Forehead	1	Angiosarcoma	Angiosarcoma	5 (3)	0/15/21
36	F	95	Right cheek	1	Subcutaneous tumor	Malignant tricholemmoma	1	0/0/49

The most rapid response was in 18 min. Twenty cases involved neoplastic disease, and 16 involved non-neoplastic disease.

*D*, days; *Hr*, hours; *Min*, minutes.

### Questionnaire survey

**Figure 3:**
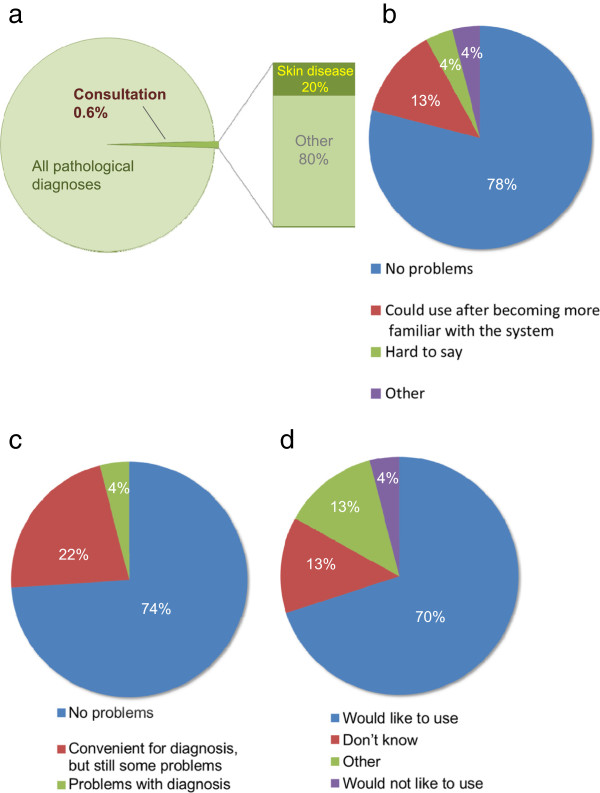
**Questionnaires and answers about our consultation system. a**) Incidences of dermatopathological consultation in Japan. Although consultations for pathological diagnosis are not so large in number, they include cases with skin disease. **b**) VS operability. Seventy-eight percent of respondents did not report any problems operating the system, and 13% answered that images could be utilized for consultation after they became accustomed to using the system. **c**) Image quality of VS. Seventy-four percent answered that image quality was sufficient for diagnosis. **d**) For future usability in consultations. Most consultants (70%) answered they would like to use this system in their work, while only 4% stated they would not.

The questionnaire was answered by 54.8% of participants (23/42). Among all routinely diagnostic cases, 0.6% cases were transferred to experts inside Japan. Of those, 20% were of dermatological disease (Figure 3a). Concerning evaluation of VS, 78% (18/23) answered that there was no difficulty with operability, and no experts noted poor operability (Figure 3b), while 74% (17/23) considered that the image quality of VS was suitable/for diagnosis (Figure 3c). Seventy percent of all participants (16/23) answered that they were interested in using this system themselves for work requiring consultation (Figure 3d). Many pathologists/dermatopathologists answered that this system is convenient as it eliminates the need to package and send prepared slides, several consultants can be involved at the same time, and prompt responses can be obtained.

## Discussion

Telemedical applications in the field of dermatology have been attempted in various ways. In 1995, Perednia et al. proposed teledermatology, in which dermatological macroscopic findings were captured by digital camera, and sent remotely via the Internet from personal computer for consultation [15]. The usefulness and some associated problems of this system have been reported [16-18]. The subsequent widespread use of digital devices and personal computers, together with the development of telecommunications, has led to the development [19] and gradual popularization [20-22] of teledermatopathology, which involves the digitization and remote transmission of microscopic dermatopathological findings. The use of dermatological consultation based on VS has been reported by Massore et al. [8,12], followed by Mooney et al. [9].

The application of telediagnosis in the field of pathology is relatively advanced, and significant contributions to the development of telepathology have been made by Weinstein et al. in the USA beginning in the early 1980s [23-25], Kaiser et al. in Europe [26,27], and by Sawai et al. in Japan from the early 1990s [28]. Along with the shift from static to robotic images and from analog to digital lines, recent developments in IT led to the emergence of the worldwide use of VS, especially in the fields of diagnosis and education [29,30], followed by diagnostic developments such as automated diagnosis of histological screening via the Internet [31]. Furthermore, efforts have been made to reduce image data volume as much as possible [32].

Thirty or more companies around the world now deal with VS systems, and several recent devices have been rated as having rapid scanning capabilities and high image quality [33].

With the increased availability of VS, many institutions have begun applying VS for telepathology. Furthermore, modern high-volume and high-speed communication lines facilitate the use of high-resolution WSI. However, in terms of magnification, focus adjustment, and selection of specific points on a slide, diagnosis using WSI still cannot match the performance achieved with a microscope and glass slides [34-36]. However, our system is far more convenient than conventional microscopes and slides in terms of the time and manpower required, and shipping costs are avoided, although of course initial set-up costs are incurred.

Many medical universities and laboratory institutes now offer consultation services using VS data [37]. The Medical Electronic Consultation Expert System (MECES) based on an open platform has performed in Europe [26]. This is an Internet communication service based on grid tehchnology [38].

Although there may be local consultation systems using static and robotic images, until now a consultation system using VS has not been available in Japan [39]. This is the first multi-function VS system equipped to handle both domestic and international consultations.

The system that we have developed not only shows VS images, but also facilitates within a single system the communication of opinions involving clinical and pathological data necessary for diagnosis. The most characteristic point of our system is that data for diagnosis involving scanned images and other related data can be sent out at the same time via the Internet to multiple consultants. One of the biggest advantages of consulting many pathologists is that we can obtain various opinions reflecting the professional field and interests of each pathologist. Using our system we consulted more than one consultant in six cases, because of uncertainty about our diagnosis, or concern over difficult cases outside our professional field. For general pathologists, diagnosis in the very specific field of dermatopathology is rather difficult compared with other fields such as diseases of the gastrointestinal tract and respiratory tract. So far we have not changed our diagnosis on the basis of replies from consultants, but when consultants returned replies consistent with our own diagnosis, this gave us added confidence to communicate the diagnosis to the clinician. For this reason, this system could be particularly helpful for pathologists working alone in a single institute. Even if our diagnosis could not consistent with the diagnoses of consultants, we could learn much about the process of how to determine the diagnosis, and could obtain new knowledge from experienced pathologists. In the case of differences in diagnosis, we can consider the opinions of other consultants, but we must take final responsibility for making a diagnosis and informing the clinicians.

This system is much faster, more economical, and more convenient than the conventional method of directly mailing glass slides. Until now it required from 5 to 10 days to receive a diagnosis because of the delays associated with preparing, packaging and sending slide glasses by mail. In our VS system the fastest diagnosis was returned only 18 minutes after sending. Quick responses are obviously helpful for patients, as well as for pathologists and for clinicians who must administer therapy. The waiting time until therapeutic decisions are made is a big source of stress for patients. Quick diagnosis using telepathology relieves this stress and so gives satisfaction to patients as well as to pathologists.

This system is now in development for practical application overseas. Despite time differences, the answer from Yanbian University in China was returned only 16 hours after sending the request. Although it sometimes took several days to receive a response, this delay mainly resulted from consultant dermatologists and pathologists being too busy to log on to their personal computers (personal communication), or from cases for consultation being fairly difficult even for specialists and thus requiring time for investigation. Delays were thus probably not due to the poor quality of the consultation system, but to factors related to rare cases or to the consultants’ busy schedules.

**Figure 4:**
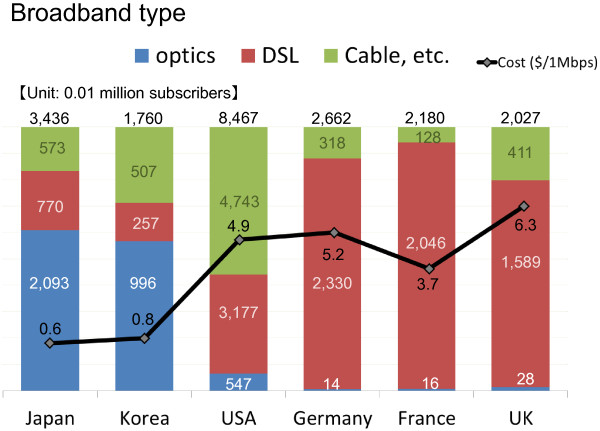
**Prevalence of broadband (Ref.**[40]**,**[41]**modified).** High-speed and high-capacity fiber-optic communications have been spreading throughout the world. There are differences between countries in the kind and level of infrastructure. Japan had a lower cost per 1Mbps compared with other countries.

The VS data ranged in size from 150 MB to 300 MB, far greater than that in the case of static images. Therefore, a high-performance personal computer and high-speed telecommunication lines are required for rapid monitor display. The infrastructure in Japan is characterized by the wide availability of inexpensive, high-speed broadband (Figure 4) [40,41] and offers an environment well-suited to mass data transmission and reception.

Major advantages of this system are that the integration of a VS system and a consultation web application allows diagnoses to be requested via only a web browser, without depending on a specific operating system or specific software; up to 5 VS images can be sent at a time; and requests for diagnosis can be sent to up to 10 diagnosticians within and outside Japan at the same time (Figure 2), and the consultants can do everything from accessing the data to making the diagnosis and providing a response, anywhere and at anytime, using only a standard personal computer and public networks. Thus, the system is advanced in that the entire procedure can be managed on a web browser through a personal computer, from the preparation of the digital images and patient information and transmission of image data, to the retrieval of data and the transmission of answers by the receiver side, and again in the verification of the response on the sender side. The results of the questionnaire showed that more than 70% of consultants felt there were no problems with the quality of VS images and operability (Figure 3b, c), and that 70% were interested in using the system for their own work (Figure 3d).

Making our consultation system more widely available in the future will require more powerful personal computers, lower costs, and provision of financial resources for payment for diagnosticians, as well as public insurance coverage, standardization of formats of VS data, and more widespread use of high-speed telecommunication lines.

The ramifications of crossing national boundaries also remain to be considered. Our study is still a pilot study aimed at improving usability and overcoming any other problems with using this system. If the system is well received, we will then have to consider problems such as licensure, quality control, billing and medico-legal liability across national boundaries.

We nevertheless hope to be able to implement the system on a wider basis both to facilitate conventional consultation about difficult and uncertain cases, and to better facilitate the establishment of international diagnostic criteria by increasing international collaboration between many specialists without the need for the time and cost associated with participation in international conferences and other gatherings. However, the wider implementation of this system depends crucially on the level of IT infrastructure and the specifications of personal computers. With continued progress in IT infrastructure, we believe that many pathologists throughout the world will be able to apply this system for diagnosis.

## Conclusion

We have developed a novel teledermatopathological consultation system using VS, and investigated the operability, convenience, and image quality when attaching the necessary data related to diagnosis and transferring them via fiber-optic lines to consultants. The results demonstrated that our consultation system is a useful tool not only for dermatopathology but also for clinical dermatology in the future. However, the current study had its limitations, and in future we hope to conduct a more rigorous study into the effectiveness of our system compared with conventional glass slides, and with other VS systems.

## Competing interests

The authors declare that they have no competing interests.

## Authors’ contributions

Performed the experiments and wrote the paper: TM and IN. Revised the paper: AK and MU. Supervised the developer of the system: KS. Assisted with writing the manuscript: JH. Participated the experiments: TA. Participated in the conception of this study, designed the experiments and final approval of the article: TS. All authors read and approved the final manuscript.

## Authors’ information

First author: Ikunori Nakayama.

Co-first author: Tsubasa Matsumura.
